# A case study of abdominal wall and limb necrotizing fasciitis: an extremely rare post -operative complication

**DOI:** 10.1186/s12905-024-03084-1

**Published:** 2024-04-15

**Authors:** Saida Sakhri, Ons Krimi, Nayssem Khessairi, Fethia Abidi, Maher Slimane, Hanen Bouaziz, Tarek Ben Dhiab

**Affiliations:** 1 Department of Surgical Oncology, Faculty of Medicine of Tunis, University of Tunis El Manar, Salah Azaiez Institute, Boulevard 9 Avril 1938, Tunis, Tunisia; 2 Department of Radiology, Faculty of Medicine, Salah Azaïz Institute, University Tunis El Manar, Tunis, Tunisia

**Keywords:** Cervical cancer, Post surgical complication, Abdominal wall, Necrotizing fasciitis

## Abstract

**Introduction:**

Infectious affections are the most frequent post-operative complications, the rate have been reducing due to the administration of perioperative antibiotics and they are rarely serious. They are usually associated to pelvic collections, fistulas, urinary tract stenosis and, exceptionally, necrotizing fasciitis (FN) and pelvic organ necrosis. There is no well-codified treatment.

**Case presentation:**

A 42-year-old female patient, was referred to our department for a stage IIIC2 adenocarcinoma of the uterine cervix. Two months after surgery, the patient presented with fever. Abdominal CT scan revealed a recto-vaginal fistula. The patient underwent a surgical evacuation of the collection and a bypass colostomy.

Post-operative period was marked by the occurrence of an extensive necrosis to pelvic organs and medial left leg’s thigh compartments muscles. She also presented a thrombosis of the left external iliac vein and artery.

Given the septic conditions, a revascularization procedure was not feasible. A bilateral ureterostomy was required and a ligature of the left external iliac vessels. Then she received palliative treatment.she died one month after surgery because of multivisceral failure due to sepsis.

**Conclusion:**

Necrotizing fasciitis is extremely rare and serious condition, the diagnosis is clinical and radiological, CT scan is helpful for the. There are predisposing factors such as diabetes, neoadjuvant radiotherapy or chemotherapy. The prognosis can be improved with rapid management and appropriate medical and surgical excisions of necrotic tissue, and antibiotic therapy adapted to the suspected germs, essentially anaerobic ones.

## Highlights

• Necrotizing lesions of softs tissus are an extremely rare complication in routine surgical practice.

• The diagnosis of necrotizing fasciitis is difficult and based on clinical symptoms, the particularities of the mixed infection, and predisposing factors of the patient.

• The Treatment is based on an urgent and intensive surgical debridement with targeted application of broad-spectrum antibiotics.

## Introduction

Infectious affections are the most common postoperative complications but rarely serious, rates have recently decreased due to the administration of perioperative antibiotics. They are usually associated with pelvic collections, fistulas, urinary tract strictures and, exceptionally, necrotising fasciitis (NF) and pelvic organ necrosis [1].

The term "necrotising fasciitis" include several syndromes of progressive gangrenous infection of the skin, subcutaneous tissue, and fascia. It is a rare and rapidly progressive infection which can be associated with thrombosis, muscle destruction and liquefaction of fats [2, 3].

There were some predisposing factors led to the development of infection, such as diabetes, body mass index greater than 40%, an immunocompromised status, previous treatment by radiotherapy, chemotherapy or pelvic surgery. And may be the absence of prophylactic antibiotic therapy during primary surgery. The etiological agents are aerobic and anaerobic bacteria such *as Aerococcus viridans, Peptostreptococcus spp, Enterococcus faecalis and Escherichia coli *[3].

There is no well codified therapeutic consensus, treatment must be individualized according to the patient's morbidities and the particularities of each case. Urgent treatment of pelvic necrosis requires surgical debridement of necrotic tissue with a broad-spectrum antibiotic. Delay in primary surgical debridement is the most important prognostic factor affecting survival.

Here we present a rare case of necrotizing fasciitis of the abdominal wall and extremities, which developed after surgery for cervical cancer.

So far this is the first case reported in our institute, either in the literature there were some studies reporting this complication, the peculiarity of this case is the aggressiveness of this complication and the delay in diagnosis and treatment.

The aim of our study is to emphasize the seriousness of the infection and the importance of early diagnosis. We have tried, through our case and through the literature, to clarify the standard treatment.

### Case presentation

We report the case of 42-year-old woman with no personal or familial history and no medical comorbidities, who was referred to our department for a stage IIIC2 adenocarcinoma of the uterine cervix. She underwent four courses of chemotherapy based on cisplatine combined with pelvic radiotherapy at a dose of 45 Gy, complicated by post radiation rectitis and cystitis. She did not receive brachytherapy for being beyond the deadline. Evaluating pelvic magnetic resonance imaging (MRI) showed a good therapeutic response associating tumor size regression (32 mm VS 40 mm), disappearance of parametrial involvement and lymph node invasion. The patient underwent colpohysterectomy. The immediate postoperative course was uneventful, and the patient returns home four days after surgery.

Final histological examination concluded to a 10 mm remnant of a cervical HPV positive adenocarcinoma. Surgical limits and parameters were free of tumor. The multidisciplinary committee of our institute did not indicate any adjuvant therapy. Two months after surgery, the patient presented to the emergency department because of fevers of 39 °C, pelvic pain, and painful mobilization of the left lower limb. The patient did not have any primary or acquired immunodeficiency. An abdominal CT scan revealed a recto-vaginal fistula fed by a 5 cm pelvic collection originating from the uterine cavity. She was admitted and treated with antibiotics and then underwent surgical evacuation of the collection and a bypass colostomy.

**Fig. 1:**
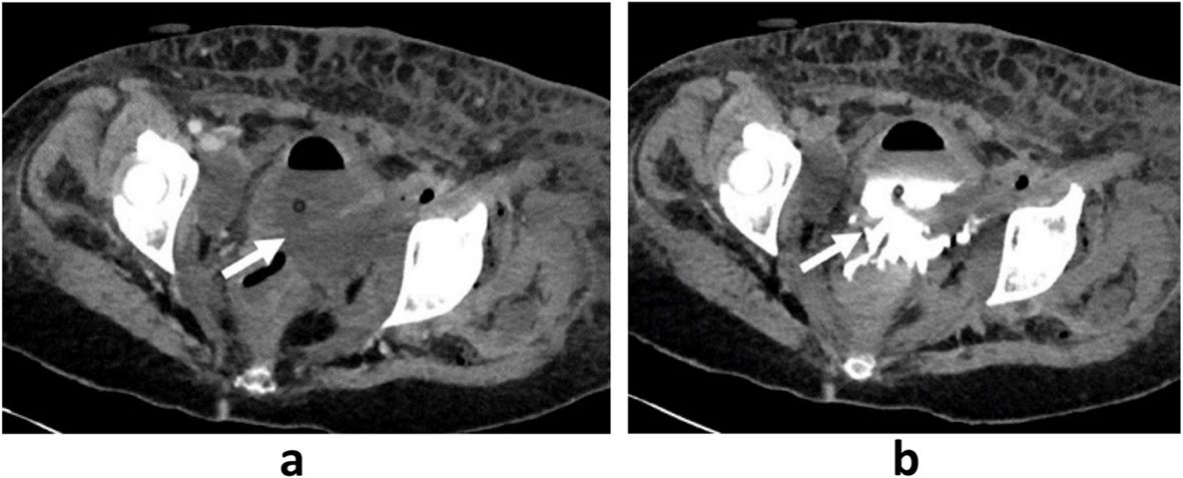
Axial section of a CT scan injected at the venous (**a**) and excretory (**b**) phase: rupture of the bladder wall (arrow)

**Fig. 2:**
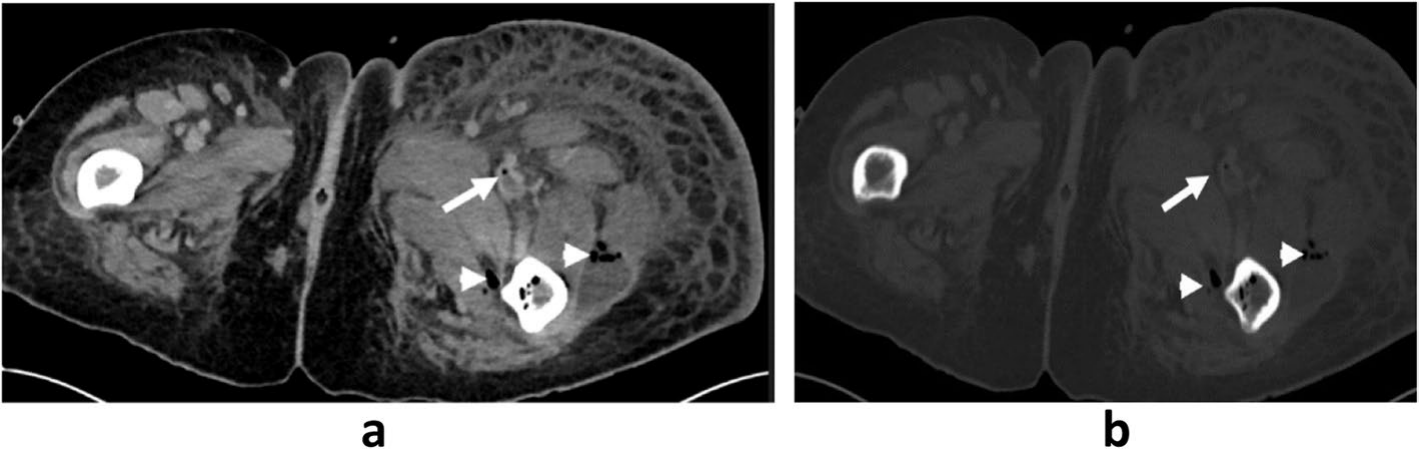
Axial CT section injected in the abdominal and bone window showing thrombosis of the superficial femoral artery seat of air bubble (long arrow) and intramuscular collection seat of air bubble (short arrow)

**Fig. 3:**
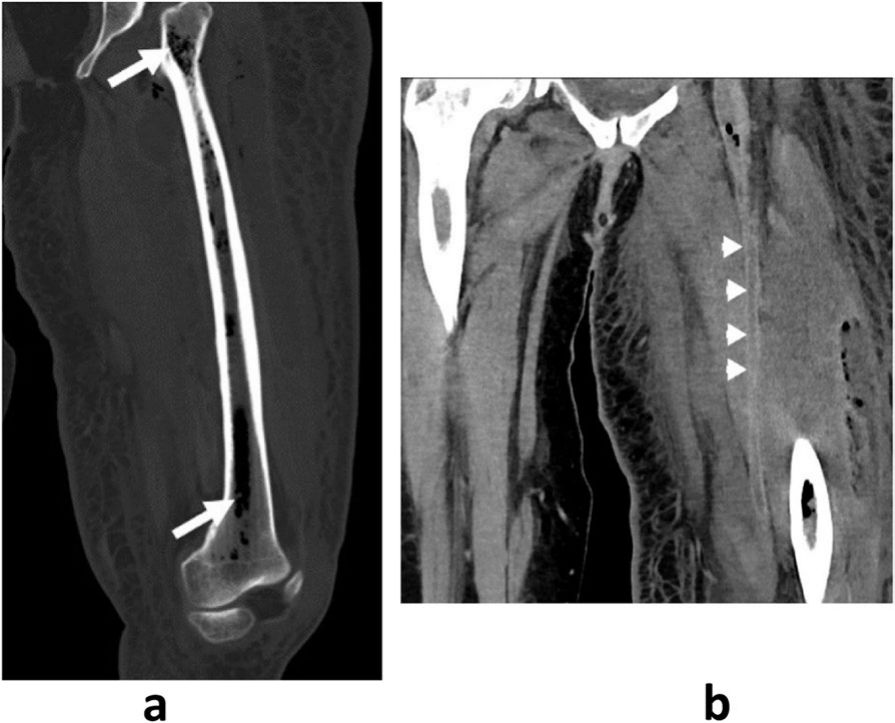
Oblique Coronal reconstruction in the bone window (**a**) and oblique coronal along the femoral artery reconstruction (**b**): pan-diaphyseal air bubble of the left femoral diaphysis (long arrow) thrombosis of the artery superficial femoral (short arrow)

The initial progression was favorable, but within two weeks, the patient presented a rise of temperature with swelling and recurrence of pain on mobilization of the left lower limb. Clinical examination found a swollen, edema, hot skin and “crackling snow crepitus” among the left lower limb. No bullae or other skin signs predicting necrosis were appreciable. An urgent computed tomography (CT) scan of the chest, abdomen, and pelvis showed a 10 cm pelvic collection lateralized to the left and fistulated into the bladder and rectum. It also showed intramuscular collections, pan-diaphyseal air bubbles of the left femur with a myositis-like appearance of the gluteal muscles, anterior and medial thigh compartments, and left leg muscles, suggestive of an infectious origin. Thrombosis of the left external iliac artery and left femoral vein were additionally noted (Figs. 1, 2 and 3).

**Fig. 4:**
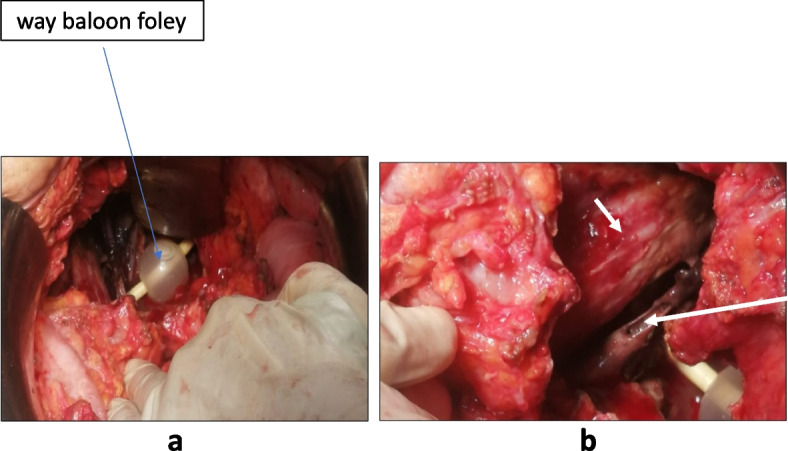
**a** operative imaging showing an extensive necrosis to the bladder and the anterior wall of the rectum. **b** extensive necrosis to pelvic organs and the psoas muscle (short arrow). Thrombosis of the left external iliac vein and artery, with necrosis of their vascular walls (long arrow)

**Fig. 5:**
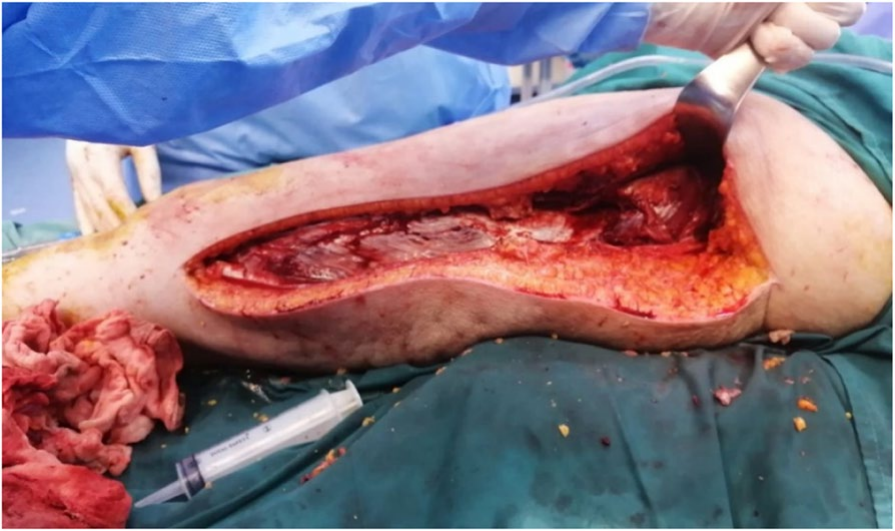
Per-operative imaging showing an extensive necrosis to the anterior and medial thigh compartments muscles of the left leg

The patient was then re-operated; the intraoperative exploration found an extensive necrosis to pelvic organs namely the bladder and the anterior wall of the rectum which extended to the anterior and medial thigh compartments muscles of the left leg. We also noted a thrombosis of the left external iliac vein and artery, with necrosis of their vascular walls Figs. 4 and 5.

Given the septic conditions, a revascularization procedure was not feasible. A bilateral ureterostomy was required, as well as ligature of the left external iliac vessels. Necrotic tissue of the left lower limb was also effectively excised and revived. The evolution was marked by left lower limb loss of functionality, despite the development of collateral circulation. The patient underwent post-surgical palliative treatment, and she died one month after surgery of multivisceral failure due to sepsis.

## Discussion

The term of Necrotizing fasciitis (NF) was firstly described by Jones in 1871 and it was firstly defined as hospital gangrene, other authors grouped this clinical entity in a single category of progressive necrotizing infections. It is a rare and rapidly progressive gangrenous infections of the skin and subcutaneous tissue [3]. Usually associated with thrombosis due to the necrosis of skin and soft tissue, and destruction of muscles [3, 4].

Later, in 1952, Wilson used this term and suggested that aetiological factors such as surgical treatment [3]. chemotherapy and radiotherapy can rarely induce necrosis of pelvic organs. The necrosis can be explained by cellular depletion, epithelial atrophy as well as hypovascularisation leading to tissue hypoxia and micro-ulcers that can lead to fistula development and make the area susceptible to infection, pelvic collections, and necrosis a few months after radiotherapy [4, 5].

Nakano et al. reported an incidence of late complications of pelvic radiation in the urinary tract as 18% in 1148 patients treated with exclusive pelvic radiotherapy. According to this study, toxicity depends on the radiation dose, treatment volume and tumor extension [4]. This hypothesis seems to be more acceptable for our case, in fact the patients received neoadjuvant treatment that contributed to rectovaginal, and vesico-vaginal fistulas, as well as post-operative collections and abscesses, and finally necrotizing fasciitis.

Classic clinical presentation involving the triad of fever, abdominal or spinal pain and painful limitation of lower limb’s movements is present in only 35% of cases. NF is masked in 85% by abscess or cellulitis, which explains the frequently delayed care [5].

The skin appeared swollen and decolorated and a sense of crackling snow suggests the presence of subcutaneous gas. The progression is marked by the development of tense edema, necrosis, and crepitus that are major signs of necrosis. However, hemorrhagic bullae and crepitus are sinister signs, with the likelihood of underlying fascia and muscle being compromised [5].

Without treatment and in some times, despite administration of intravenous antibiotics, the progression of the disease among the tissues continues and severe general symptoms occur like tachycardia, fever, altered mental state, and diabetic ketoacidosis [5].

CT imaging is a helpful tool in distinguishing NF from other infections of the soft tissues such as cellulitis, soft tissues abscess and osteomyelitis which require different management [5]. It can demonstrate skin thickening, septation of the subcutaneous fat, and thickening of the underlying superficial fascia. Necrosis appears on the CT scan as a lack of the enhancement of the fascia as well as thickening of the skin and underlying superficial fascia [2]. MRI has no place on first intention,it can be useful to evaluate the exact extension of the infection to the surrounding organs and to distinguish mild fascial or muscle involvement [5].

In the present case rectal fistula caused intra-abdominal infection which spread from abdominal wall to the limb by contiguity, invading soft tissues (basically along the psoas muscle) or through pelvic orifices (inguinal canal, obturator foramen).

The involvement of the limb's deep fascia is rapidly progressive, potentially leading to necrosis of adjacent tissues. This complication may require sacrifice of the limb and can be fatal even after adequate management. Treatment usually involves extensive excision of necrotic and inflammatory tissues, metabolic changes, such as hyperglycemia and keto-acidosis, shsould also treated, combined with intravenous antimicrobial therapy (Penicilin G, Clindamycin, Imipenem, Teicoplanin) [6]. Mortality of untreated cases may reach 100% [3, 7].

Iliac vessel thrombosis is a rare complication of FN, it may be due to tumor mass effect or to the infection. It has no specific symptoms. CT scan confirm the diagnosis and assessing the extent of lesions [8]. The venous and arterial thromboses observed in our patient's case can be explained by venous stasis due to vascular compression by the voluminous collection, endothelial lesions engendered by the inflammatory and septic environment, and post-radiation fibrosis phenomena altering the vascularization of the pelvic organs, leading to necrosis of the vascular walls and bladder [9].

In our case the bladder necrosis can be explicated by radiotherapy which caused fistula and spread to FN. In the literature post radiation necrosis of the bladder has been described in few studies; Marnitz et al. reported a case of uterine necrosis after radiotherapy and chemotherapy for cervical cancer and a case of uterine and bladder necrosis requiring anterior exenteration [9].

Also Micha et al. reported a case of extensive pelvic necrosis in a patient treated for cervical cancer and surgery followed by radio-chemotherapy requiring multiple and repeated surgery over 15 years [10]. Also, Sanna et al. described a case of extensive pelvic necrosis after chemoradiotherapy for locally advanced cervical cancer [11].

Rebai et al. reported a rare case of FN of chest and right abdominal wall caused by acute perforated appendicitis treated with intravenous antibiotics and surgical debridement oxygenation of infected tissue. Unfortunately, on postoperative day, the patient developed a mitral valve infective endocarditis and he died of septic shock and multiple organ failure [12].

If FN affects the extremities, amputation is required. However, if intra-abdominal organs are involved, early recognition requires aggressive surgical debridement of necrotic and non-viable tissue and should be performed urgently if there is a strong clinical and radiological suspicion of necrosis [5, 13].

Some author recommanded after the initial prompt diagnosis, resuscitation and surgical debridement, re-look surgery should be considered after 24–48 h to reassess and re-debride if indicated [14].

An optimal timing for the debridement is within six hours after the onset of clinical symptoms, however treatment delay significantly influences patient’s survival. The nature of the surgery depends on the extension of necrosis [5]. Initial administration of intravenous broad-spectrum antibiotics coverage should be started before surgery and should cover pyogenes, aureus, and Gram-negative aerobes and anaerobes. Also anticoagulation in case of thrombosis should be maintained for at least three months [3].

Bladder necrosis was a real therapeutic challenge: only one case of complete recovery from subtotal bladder necrosis has been reported in the literature, requiring repeated cystoscopy to excise the necrotic tissue [15]. To achieve this attitude, we should have a partial necrosis of the bladder mucosa, sparing the detrusor, to allow regeneration of the mucosa. otherwise, bladder reconstruction is the only alternative, which was not feasible for our patient given the septic conditions and fragility of the bladder wall post radiation [16]. Hyperbaric oxygen has also been used as an adjunct to surgery and antibiotics helping to heal and regenerate tissues after debridement, improve vascularization and reduce bacterial colonization [7, 13].

The rapid progression of necrosis explains the poor prognosis of the disease, as there is little chance of complete debridement, and surgery is usually limited by septic shock and organ failure, which can rapidly lead to death, particularly in untreated patients [5].

In our case, there was a delay in diagnosis and treatment; in fact the early stages of the disease are often misdiagnosed due to the absence of specific clinical features that explain the progression of the disease and the patient's death. The lesson to be learnt from this study is the importance of early diagnosis and the usefulness of biological tests and radiology. Surgery is the gold standard because inadequate surgical intervention has a significant impact on mortality. Treatment of NF includes early surgical debridement and broad-spectrum antibiotics. The mortality rate in these cases is between 50 and 80%. without surgery, the mortality rate approaches 100%, depending on morbidity factors such as renal insufficiency, respiratory distress syndrome and multi-organ failure, as well as the speed of surgical incision. It has been shown in the literature that a delay in surgery of more than 24 h is an independent risk factor for mortality [3, 7].

## Conclusion

Necrotizing fasciitis is a gangrenous infection of the skin, subcutaneous tissue, and fascia. There are predisposing factors such as diabetes, neoadjuvant radiotherapy, or chemotherapy. Diagnosis is clinical and radiology is helpful in diagnosis and in distinguishing FN from non-FN infections. The urgent need is to differentiate NF from non-necrotising fasciitis and other musculoskeletal infections and to assess disease extension. Standard treatment is based on early recognition and extensive surgical debridement of necrotic and non-viable tissue.

In this study we present a rare case of NF, unfortunately the diagnosis was made late which had a negative impact on survival, the patient did not respond to treatment despite surgical excision and antibiotic therapy.

The Strengths of our study is the clinical and radiological particularity of this case, which remains rare but with high morbidity and mortality. This case gives as the opprtunity to evoid the misdiagnosis so we can provide a well-codified treatment. However, the weakness this study is that it is a case report so we can't generalize the results, also the lack of bacteriological analysis data of the patient.

Although the rarity of this complication, a high index of suspicion is required, especially in immunocompromised patients, and immediate treatment with urgent surgical debridement and broad-spectrum antibiotic therapy is essential to prevent further damage. Complete excision of the necrotic tissue is the only cure for this deadly infection.

## Data Availability

The datasets used and/or analysed during the current study available from the corresponding author on reasonable request.

## Abbreviations

FN: necrotizing fasciitis
CT: Computed tomography
MRI: magnetic resonance imaging
